# Intercellular Communication in Tumor Biology: A Role for Mitochondrial Transfer

**DOI:** 10.3389/fonc.2018.00344

**Published:** 2018-08-28

**Authors:** Patries M. Herst, Rebecca H. Dawson, Michael V. Berridge

**Affiliations:** ^1^Malaghan Institute of Medical Research, Wellington, New Zealand; ^2^Department of Radiation Therapy, University of Otago, Wellington, New Zealand; ^3^School of Biological Sciences, Victoria University of Wellington, Wellington, New Zealand

**Keywords:** cancer, mitochondria, intercellular transfer, stress, damage, treatment-resistance

## Abstract

Intercellular communication between cancer cells and other cells in the tumor microenvironment plays a defining role in tumor development. Tumors contain infiltrates of stromal cells and immune cells that can either promote or inhibit tumor growth, depending on the cytokine/chemokine milieu of the tumor microenvironment and their effect on cell activation status. Recent research has shown that stromal cells can also affect tumor growth through the donation of mitochondria to respiration-deficient tumor cells, restoring normal respiration. Nuclear and mitochondrial DNA mutations affecting mitochondrial respiration lead to some level of respiratory incompetence, forcing cells to generate more energy by glycolysis. Highly glycolytic cancer cells tend to be very aggressive and invasive with poor patient prognosis. However, purely glycolytic cancer cells devoid of mitochondrial DNA cannot form tumors unless they acquire mitochondrial DNA from adjacent cells. This perspective article will address this apparent conundrum of highly glycolytic cells and cover aspects of intercellular communication between tumor cells and cells of the microenvironment with particular emphasis on intercellular mitochondrial transfer.

## Introduction

Tumor development depends critically on the intimate interplay between individual neoplastic cells, normal cells from the tissue of origin, and their abiotic environment. This concept, originally proposed by Paget (1) in his “seed and soil” analogy, is now well-established. In the last few decades, the focus has been on cumulative driver mutations and loss of suppressor gene function in cancer cells. However, it is the microenvironment of the developing tumor that acts as the natural selector, resulting in expansion of the best adapted (“fittest”) clones over time (2–4, 5). Tumors can therefore be seen as evolving clones of cancer cells within an increasingly disorganized tissue microenvironment that compete for resources and are characterized by an evolving set of hallmarks (6). Individual tumors are made up of cancer stem cells with self-renewal and multiple differentiation properties, and proliferating progenitors with limited differentiating potential alongside normal tissue cells (7). The tumor microenvironment consists of cells from the tissue of origin, activated fibroblasts, invading immune cells and vascular cells, embedded in an extracellular matrix (ECM) that contains various connective tissue structures as well as growth factors, cytokines and chemokines, metabolites and nutrients, electrolytes, oxygen, etc. [reviewed by Kalluri (8)]. Infiltration of the tumor by stromal cells and immune cells can either promote or inhibit tumor growth, depending on the cytokine/chemokine milieu of the tumor microenvironment and its effect on cell activation status. Recent research has shown that stromal cells can donate healthy mitochondria to respiration-deficient tumor cells, restoring normal respiration as well as their ability to form tumors in mice. This perspective article will cover aspects of intercellular communication between tumor cells and cells from the tumor microenvironment with particular emphasis on intercellular mitochondrial transfer.

## Cells in the tumor microenvironment

Within the developing tumor, activated fibroblasts generate connective tissue that structurally supports the tumor as it grows first at its primary site and later during metastasis. Fibroblasts are extremely resistant to various stressors, including cancer treatments like radiation, and chemotherapy. Normal tissue stroma contains few fibroblasts that are in a resting state with basal metabolic activity. Following tissue injury, resting fibroblasts become activated, contractile, highly proliferative, and migratory. They produce growth factors and cytokines that recruit immune cells, promote angiogenesis, and remodel the extracellular matrix by altering connective tissue components. In the context of acute injury, activated fibroblasts facilitate wound healing, and tissue regeneration and return to their resting state after repair is complete. The presence of cancer cells within a tissue ecosystem results in fibrosis, a chronic wound healing response mediated by stromal cells in the developing tumor [see Kalluri (8)]. These activated fibroblasts called cancer- or tumor-associated fibroblasts, referred to here as CAFs, can also be recruited to the tumor by growth factors released by cancer cells and infiltrating immune cells. Several recent reviews cover various aspects of the rapidly expanding CAF literature, including their origin, activation, recruitment, interactions with tumor cells and immune cells, and role in treatment resistance (9–12). CAF precursors include resident tissue fibroblasts, bone marrow-derived mesenchymal stem cells, hematopoietic stem cells, epithelial cells (via mesenchymal-epithelial transition), and endothelial cells (via mesenchymal-endothelial transition). Most CAFs express α-smooth muscle actin (α-SMA), fibroblast activation protein (FAP), and platelet-derived growth factor receptor (PDGFR)-α and -β. CAFs promote tumorigenesis in various ways, e.g., though secretion of growth factors and cytokines, and the degradation of ECM proteins. Activation into CAFs is accomplished through epigenetic alterations, changes in the expression of non-coding miRNAs and long non-coding RNAs and the aberrant activation of several signaling pathways such as NFκB, IL-6/STAT3, FGF-2/FGFR1, and TGF-β/SMAD (10, 12, 13).

Immune cells including T cells, macrophages and dendritic cells recruited by IL-1α and the epithelial chemokine TSLP (14), infiltrate the developing tumor through the highly permeant vasculature and via migratory processes, and promote or inhibit tumor progression by generating pro- and anti-inflammatory responses or mediating immune attack, depending on the mutational load of the tumor and other factors (15, 16). Some CAFs are highly immunosuppressive and can protect the tumor from immune attack. Costa et al. (11) very recently demonstrated that only myofibroblast CAFs that express FAP (CAF-S1) were strongly immunosuppressive and these cells were found to be particularly enriched in triple negative breast cancers. In contrast, a low α-SMA-expressing subpopulation of CAFs in mouse and human pancreatic ductal adenocarcinoma were highly pro-inflammatory, producing high levels of IL-6 which stimulated tumor growth via STAT3 activation (17).

**Figure 1:**
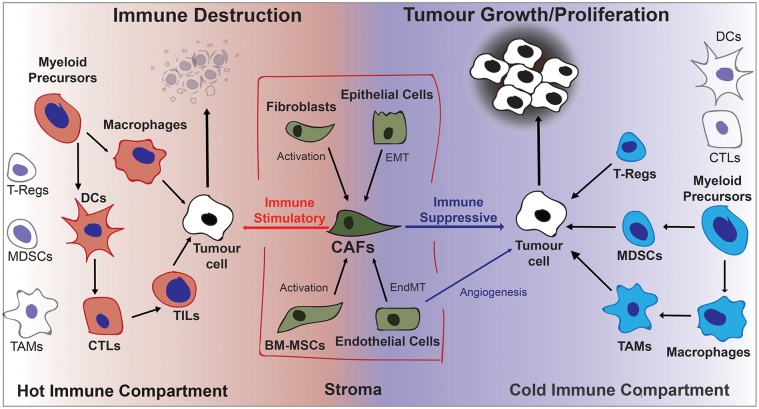
Diagrammatic overview of cells contributing to the fate of the developing tumor in the tumor microenvironment. Activated stromal cells that become CAFs and immune cells can either stimulate or inhibit tumor growth depending on the cytokine/chemokine environment. Generally a pro-inflammatory “hot” microenvironment will favor immune destruction and an anti-inflammatory “cold” microenvironment will favor tumor progression. BM-MSCs, Bone Marrow-derived Stromal Cells; CAFs, Cancer-Associated Fibroblasts; DCs, Dendritic Cells; EndMT, Endothelial-to-Mesenchymal Transition; EMT, Epithelial-to-Mesenchymal Transition; MDSCs, Myeloid Derived Suppressor Cells; TAMs, Tumor Associate macrophages; TILs, Tumor Infiltrating Lymphocytes; Tregs, regulatory T lymphocytes.

Other cells that assist tumor progression are recruited by growth factors such as vascular endothelial growth factor A (VEGFA), cytokines, and chemokines secreted by cancer cells and CAFs. Recruited endothelial cells are highly proliferative and develop leaky vascular structures that provide nutrients and oxygen to the developing tumor (8). These structures are also centrally involved in tumor metastasis that involves breaking constraints on tissue boundaries, basement membrane penetration, intravasation, circulation, extravasation, and seeding in tissues of distant organs. Figure 1 depicts the different cell types in the tumor microenvironment and their effect on tumorigenesis.

## Energy metabolism in the tumor microenvironment

The TME of most if not all solid cancers is characterized by strongly fluctuating oxygen levels with very steep and transient oxygen gradients caused by highly compromised tumor microvasculature. This challenging environment favors cells that can easily shift the balance between mitochondrial and glycolytic energy metabolism. This metabolic shift is controlled by hypoxia-inducible factor 1α (HIF-1α) which is highly expressed in most solid tumors [reviewed in Courtnay et al. (18)]. Most, but not all, highly aggressive tumors bias their energy metabolism toward glycolysis irrespective of oxygen levels, a phenomenon referred to as the *Warburg effect* (19). The balance between mitochondrial and glycolytic energy could be viewed as a “rheostat” rather than an “on/off” switch as both are essential for life in physiological situations. A rheostat strategy allows cells to finely balance their energy requirements according to oxygen and nutrient supply with glycolytic intermediates available for anabolic processes. It would also allow fast proliferating cells to escape the detrimental effects of high levels of reactive oxygen species (ROS) generated during mitochondrial electron transport whilst retaining adequate ROS levels for signaling and mitogenic purposes [reviewed in Idelchik et al. (20)]. Mutations in mtDNA, changes in mtDNA copy number and epigenetic changes to mtDNA affecting mtDNA gene expression, are very common in a large variety of different types of cancer (21) leading to a re-balancing of mitochondrial and glycolytic energy metabolism to favor glycolysis. Highly glycolytic phenotypes have been associated with increased invasive and metastatic potential and chemoresistance to cancer treatments [reviewed by Guerra et al. (22)]. In most instances cells with mutated mtDNA or reduced mtDNA copy number retain some level of functional mitochondrial electron transport. Tumor cells without any mtDNA such as ρ^0^ cells completely lack functional mitochondrial electron transport and survive *in vitro* only when supplemented with uridine and often pyruvate (23).

Based on the aggressive nature and poor patient prognosis of many highly glycolytic tumors we expected that our metastatic murine breast (4T1) and melanoma (B16) ρ^0^ cells would generate tumors at the same rate or faster than the parental cells. However, tumor cells without mtDNA produced tumors only after a long lag period compared with parental cells (24, 25). Surprisingly, these cells had taken up mtDNA (25) and therefore mitochondria (26) from cells in the tumor microenvironment of the host mouse, and had recovered respiratory capacity. These findings led us to hypothesize that purely glycolytic ρ^0^ cells cannot form tumors unless they acquire mtDNA from elsewhere. This apparent conundrum between aggressive highly glycolytic tumors and purely glycolytic ρ^0^ tumor cells that cannot form tumors needs further consideration. The explanation we believe lies in the detail: highly glycolytic cells likely have some respiratory capacity, even though they may not use it or depend on it. Purely glycolytic ρ^0^ tumor cells have no functional respiratory complexes and therefore no mitochondrial electron transport, explaining their auxotrophy for uridine. This is because respiratory capacity is required for the activity of dihydroorotate dehydrogenase (DHODH), a flavoprotein found on the outer surface of the inner mitochondrial membrane. DHODH catalyzes the ubiquinone-mediated fourth step in pyrimidine biosynthesis, the oxidation of dihydroorotate to orotate. Electrons from this oxidation are used to reduce coenzyme Q (CoQ) just prior to complex III in the electron transport chain (23). In the absence of functional mitochondrial electron transport, DHODH is unable to oxidize dihydroorotate, thus blocking pyrimidine biosynthesis. Adding uridine to the growth medium bypasses the block in pyrimidine biosynthesis and thus DNA replication and is therefore required for the maintenance of ρ^0^ cells in culture (23). Other substrates such as pyruvate are needed with some ρ^0^ cells. In a nutshell, ρ^0^ cells cannot synthesize DNA and are unable to divide and therefore cannot form tumors in mice as the tumor microenvironment does not have enough uridine to support DNA synthesis. In contrast, cells with mutated mtDNA or decreased mtDNA copy number have reduced ability to use the electron transport chain and may rely on glycolytic energy production, but they are still able to synthesize pyrimidines and thus are able to form tumors *in vivo*. Some authors (22) have attributed slower tumor development of injected ρ^0^ tumor cells to their slower growth rate *in vitro*. However, most of these studies have not checked the ρ^0^ tumors for the presence of mtDNA or respiration recovery. Kulawiec et al reported that the complete absence of mtDNA conferred tumorigenicity to non-tumorigenic human breast epithelial MCF12A cells and increased the tumorigenic potential of human breast cancer MDA-MB-435 cells in a SCID mouse model (27). MDA-MB-435ρ° cells demonstrated increased invasive and clonogenic potential when cultured in the presence of uridine. Interestingly, MDA-MB-435ρ° cells started developing tumors in the flank of mice 3 weeks after injection without the lag phase we have seen in our ρ° tumor models. It would have been interesting to see whether or not the resulting MCF7ρ° and MDA-MB-435ρ° tumors had acquired mouse mtDNA from the tumor microenvironment and regained respiratory capacity as shown for syngeneic mouse models.

## Intercellular mitochondrial transport

Intercellular mitochondrial transfer involving horizontal transfer of the entire mitochondrial genome is an emerging concept in tumor biology that challenges well-established concepts (28). Successful mitochondrial transfer depends on communication between donor and recipient cells, even though at this stage these signals have not been clearly identified. In the next part of this perspective article, we will focus on mitochondrial transfer between stromal cells and mitochondrially incompetent cancer cells without functional respiration.

## Mitochondrial acquisition by cancer cells

### Mouse tumor models lacking mtDNA

Initial experiments investigating whether or not B16ρ° melanoma cells would grow as tumors in syngeneic mice focussed on tumor growth and not on mitochondrial acquisition (24). A 20-day lag to tumor growth was observed with B16ρ° cells injected into C57BL/6 mice subcutaneously, and a longer lag period was seen in NOD/SCID mice. In contrast, when B16ρ° cells were injected intravenously, no lung metastases were observed in these models. We used tumor-derived cell lines, PCR primers specific for the mitochondrial gene, *Cytb*, and antibodies against mitochondrially-encoded proteins to determine whether B16ρ° cells had adapted to growth in response to support from stromal cells in the tissue microenvironment, or had re-expressed latent mtDNA or perhaps even acquired mtDNA from other cells (25). Rigorous confirmation of the presence of host mouse mtDNA in this model, and in the 4T1ρ° mouse breast cancer model in Balb/c mice, involved the presence of stable mtDNA polymorphisms that occur between each of these tumors and the mouse from which they were derived. In both cases the mtDNA in the tumors that grew from ρ° cells contained the mtDNA polymorphisms of the recipient mouse and not the tumor, “proving” that mtDNA had transferred from recipient mouse cells. This mtDNA transfer was later shown to involve transfer of intact mitochondria from BM-MSCs by prior co-culture with these cells (26). Intercellular mitochondrial transfer was shown to be responsible for tumor growth and respiration recovery. Possible contribution of contaminating stromal cells to the acquired mitochondrial genotype was excluded by long-term culture of B16ρ°-derived cells. Stromal cells do not divide in the culture media used to grow the tumor cells, resulting in the dilution of stromal cells over time. In the case of 6-thioguanine-resistant 4T1ρ°-derived cell lines, stromal cells sensitive to this drug were eliminated in culture medium containing 6-thioguanine. In each model, cell lines were derived from subcutaneous tumors, from circulating tumor cells and from lung metastases, and in the case of 4T1ρ°-derived cell lines, from the orthotopic mammary gland site. These mouse models clearly demonstrate that mitochondrial transfer occurs subcutaneously and orthotopically in extreme models of mtDNA damage and show that the transferred mitochondria rescue respiration and facilitate tumor growth. The nature of the stromal cells donating mitochondria in these models was not addressed.

### Xenotransplantation

The ability of human osteosarcoma 143B cells without mtDNA (143Bρ° cells) to grow subcutaneously as tumors in immunocompromised Balb/c nude mice was investigated recently (29). Tumors grew slowly in 60% of mice inoculated with 10^6^ cells. After FACS-sorting to remove contaminating mouse stromal cells, tumors were found to contain low levels of mouse mtDNA, but no human mtDNA. FACS-sorted tumors cells were re-injected and were found to grow as small tumors with higher levels of mtDNA than the those in the original tumors, but 4 out of 5 of these tumors arrested at 200–300 mm^3^ and regressed because human nDNA replication factors do not recognize murine mtDNA promotors, resulting in dilution of mouse mtDNA in the human 143B cells.

Cells devoid of mtDNA are not the only cells that benefit from acquiring mitochondria. Human primary acute myeloid leukemia (AML) cells contain mtDNA but have greatly increased mitochondrial content when isolated from bone marrow. Mitochondrial transfer from bone marrow-derived stromal cells (BMSCs) to primary human AML blasts and MOLM-14 AML cells via endocytosis was demonstrated in xenografts in NOD/SCID/gamma (NSG) immunodeficient mice (30). In this study, the mouse mtDNA gene*, mt-Co2*, was present in four primary AML patient samples and in MOLM-14 cells FACS- purified from mouse bone marrow following transplantation. Treatment with cytarabine, etoposide, and doxorubicin increased mitochondrial transfer and tumorigenicity of AML cells. Similarly, Marlein et al. (31) reported NOX2-driven transfer of mitochondria from BMSCs to primary human AML cells injected into NSG mice via AML-derived tunneling nanotubes (TNTs). Mitochondrial transfer was enhanced by treatments that increase ROS levels in BMSC such as hypoxia, hydrogen peroxide, daunorubicin, and cobalt chloride. Inhibition of NOX2 by diphenyleneiodonium (DPI) or by NADPH oxidase-2-depleted AML cells inhibited mitochondrial transfer and increased mouse survival (31).

### Cells used as mitochondrial donors in co-culture approaches

The primary donor cell types used in co-cultures to investigate mitochondrial transfer to cancer cells have been bone marrow-derived mesenchymal stem or stromal cells (BM-MSCs), although MSCs can be derived from many different tissues. Because MSCs can give rise to CAFs (32), resting fibroblasts can be considered to be MSCs that become CAFs when stimulated (8). MSCs and CAFs share many important characteristics; they contain cells that are pluripotent and can differentiate into osteoblasts, chondrocytes and adipocytes and possibly also myocytes and neurons. Both MSCs and CAFs are highly migratory, and travel to inflamed and injured regions to facilitate repair (10, 12, 32). In tumors, MSCs increase the proliferation, invasion and metastatic potential of many solid tumors by inducing the epithelial-to-mesenchymal transition (EMT) in primary tumor cells (reviewed in Ridge et al. (32). BM-MSCs are able to donate mitochondria to both cancerous and non-cancerous cells (28, 33–37). However, these cells are functionally different from MSCs isolated from other tissues, which may affect their ability to donate mitochondria. Differences in tissue types and growth conditions can favor certain subpopulations and future research should characterize the MSCs used in mitochondrial transfer experiments.

The first demonstration of mitochondrial transfer to tumor cells involved co-culture of human A549 lung adenocarcinoma cells without mtDNA (ρ° cells) with human BM-MSCs (38). This seminal study showed that auxotrophy for uridine and pyruvate was lost and respiration restored in clones that had acquired mtDNA, and that the mitochondrial genotype was that of the donor BM-MSCs. Furthermore, whole mitochondria were transferred as shown by using donor cells with a DsRed2 construct containing a mitochondrial import sequence. Cell fusion was excluded as a plausible explanation of mitochondrial transfer in cell lines derived from A549ρ° cells and neither platelets nor isolated mitochondria, used by others in mitochondrial transplantation (39, 40), were able to act as mitochondrial donors in this system. Mitochondrial transfer from BM-MSC to human 143Bρ° osteosarcoma cells and cells depleted of mtDNA with rhodamine 6G has been reported, but surprisingly, no transfer was detected to 143Bρ° cybrids harboring pathogenic mtDNA mutations (41). Others have reported mitochondrial transfer from BM-MSCs to murine B16ρ° melanoma cells (26), human ovarian cancer cell lines (42), breast cancer cell lines (39, 42), human lung adenocarcinoma A549 cells and mouse LA-4 lung adenocarcinoma cells (43), and to primary human AML cells and several AML cell lines (30, 31). MSC's derived from umbilical cord Wharton's jelly (WJ-MSC) were shown to transfer mitochondria to 143Bρ° cells. Cells surviving selection in the absence of uridine and pyruvate and in the presence of BrdU to remove WJ-MSC contained mtDNA polymorphisms of the WJ-MSCs and not the 143Bρ° cells, and respiration was restored (44).

In addition to MSCs, skin fibroblasts (31, 38) and embryonic mouse 3T3 fibroblasts (43) have been used in co-culture studies as mitochondrial donors. Endothelial cells (ECs) have also been used as mitochondrial donors in co-culture with ovarian and breast cancer cell lines. Endothelial cells are abundant in the vasculature of developing tumors where they form the inner lining of newly-formed blood vessels. Combining BM-MSCs and ECs with MCF7 breast cancer cells in co-culture showed preferential transfer of mitochondria by ECs (42). Table 1 summarizes the donor and recipient cells used in the studies described above.

**Table 1:** Mitochondrial transfer to and from cancer cells *in vitro*.

**Donor cell type**	h/m^*^	**Recipient cell type**	**References**
**MESENCHYMAL ORIGIN (MSC)**			
Bone marrow (BM)	h	hA549rho0 lung adenocarcinoma	(38)
	h	h143Brho0 osteosarcoma	(41)
	h	h multiple ovarian and breast cancer lines	(42)
	h	hMDA-MB-231 breast cancer	(39)
	h	hAML blasts, CD34+ peripheral blood progenitors	(31)
	m	mB16rho0 melanoma	(26)
	h	hAML blasts and cell lines ± chemotherapy	(30)
(MS-5 cell line)	m	hAML blasts and cell lines ± chemotherapy	(30)
	m	mLA-4 lung adenoma	(43)
	h	hA549 lung adenocarcinoma	(43)
Wharton's jelly (WJ)	h	h143Brho0 osteosarcoma	(44)
**FIBROBLAST ORIGIN**			
Skin	h	hA549rho0 lung adenocarcinoma	(38)
	h	hAML blasts, CD34+ peripheral blood progenitors	(31)
3T3	h	hA549 lung adenocarcinoma	(43)
			
**ENDOTHELIAL ORIGIN**			
	h	h multiple ovarian and breast cancer lines	(42)
**OTHER**			
Jurkat T-ALL	h	hBM-MSC	(45)
hMDA-MB-231	h	hBM-MSC	(46)

* *h, human; m, mouse*.

## Mitochondrial transfer between tumor cells

Although not strictly related to mitochondrial transfer between host stromal cells and tumor cells, a number of studies have demonstrated intercellular mitochondrial transfer between tumor cells that are worth mentioning here. Of particular interest are reports that astrocytic brain tumors including glioblastomas form an interconnected network that protects from cell death and damage caused by radiation and chemotherapy (47, 48). These tunneling nanotube (TNT) and tumor microtube networks, visualized by confocal microscopy, were shown to transfer mitochondria and other organelles, vesicles, and small molecule messengers including calcium and siRNAs. A number of studies have also described intercellular mitochondrial transfer in a range of different cancer co-culture systems (42, 49–54).

## Mechanism of mitochondrial transfer between cells

The mechanisms of mitochondrial transfer between cells have been reviewed recently (37, 55–58). In cell co-culture approaches most focus has been on direct cell-cell connections referred to as TNTs, where a cell under stress or with mitochondrial damage signals for help from a potentially supportive donor stromal cell. These TNTs, and membrane conduits of larger dimensions, are characterized by cellular junctions containing connexin43 and by actin or microtubular structures that contain supporting mitochondrial transport adaptor and ATP-dependent motor proteins, similar to those described in axons of neurons [reviewed by Vignais et al. (59)]. In addition to TNTs, other vesicular structures have been described that contain whole mitochondria or mitochondrial fragments, often of poor quality with disorganized cristae and swollen organelles reminiscent of mitochondria destined for “transmitophagy,” a term coined to describe packaging of damaged or spent mitochondria in the optic nerve head and elsewhere in the brain that are destined for recycling in adjacent astrocytes (60). Jurkat cells subjected to chemotherapy offload their damaged mitochondria to BM-MSCs via ICAM-1-mediated cell adhesion (45) and similar transfer to BM-MSCs has been observed by others (39). Dysfunctional mitochondria in neurons in neurodegenerative diseases may also be able to manage faulty mtDNA by intercellular transfer of and therefore, transmitophagy of these mitochondria (60, 61). Cell-cell contact has also been implicated as a mechanism of mitochondrial transfer between stromal cells and AML cells (30) where an endocytic pathway was involved.

Except for astrocytoma growth in the brain of mice (47) where intravital confocal microscopy was employed to visualize mitochondrial transport between tumor cells, the mechanism of mitochondrial transfer between tumor and stromal cells has not been elucidated in tumor models *in vivo*. Isolated mitochondria have been shown to be taken up by some cell types (40). McCully et al describes transfer of isolated mitochondria into cardiac muscle cells as *mitochondrial transplantation*. Cardiomyocytes sustain mtDNA damage after ischaemic injury and decrease their ATP production leading to loss of function. Mitochondria isolated from skeletal muscle cells injected intravenously or directly into the heart muscle of the same animal, result in cardiomyocyte recovery and improved function in animal studies and in an early human study with very young pediatric patients (40).

## Visualising mitochondrial transfer

Visualising and measuring mitochondrial transfer can be challenging (62). Mitochondria-targeting fluorescent dyes (MitoTracker) can be used for short-term *in vitro* studies under defined conditions as they require a mitochondrial membrane potential, tend to leak out of mitochondria over time and can be toxic when used at concentrations exceeding manufacturer's recommendations. Mitochondrially-imported fluorescent proteins such as mitoGFP, mitoRFP, mitoYFP, and mitoDsRed are a less toxic. However, the exact location of newly acquired mitochondria within recipient cells needs to be confirmed by high resolution confocal Z-stack imaging with appropriate deconvolution strategies to exclude the possibility that mitochondria are attached to the outside of the recipient cell. Genetic approaches that use the presence of unique mtDNA polymorphisms between co-cultured cells or in tumor models provide the most convincing evidence of mitochondrial transfer. Different mtDNA polymorphisms can be quantified using qPCR or other mtDNA polymorphism amplification methodologies. Genetic approaches can also be used to study the long-term consequences of mitochondrial transfer such as in bone marrow and organ transplantation and in tumor biology where inherent mitochondrial damage is often a key feature. Combining mitochondrial genetic markers with fluorescent visualization strategies that assess mitochondrial network morphology as well as functional evaluation of the respiratory capacity of recipient cells provides the best evidence for mitochondrial trafficking between cells (62).

## Targeting cellular interactions

Combining therapies that target tumor cell-stromal cell interactions with treatments that specifically target cancer cell mutations may well be an approach of future anticancer regimens. Blocking the immunosuppressive effects of FAP-expressing CAFs increases effector T cell recruitment and function and reduces tumor growth in mice (63), and are well-tolerated in patients with advanced/refractory mesothelioma (64). Similarly, preventing mitochondrial transfer between tumor cells and donor cells within the TME that restore the tumor's respiratory ability would also augment more traditional therapies and/or restore sensitivity to radiation and chemotherapy. For example, blocking TNT formation by Cytochalasin B decreased but did not fully inhibit mitochondrial transfer from MSCs to macrophages to improve their phagocytic and bacterial clearance ability in ARDS (34), or to lung endothelial cells to attenuate cigarette smoke induced damage in COPD (35). Blocking the formation of new TNTs by Cytochalasin B did little to destabilize or destroy existing TNTs which facilitate mitochondrial transfer between rat pheochromocytoma (PC12) cells (49). Inhibition of NOX2-mediated mitochondrial transfer by diphenyleneiodonium (DPI) and antioxidants such as N-acetyl cysteine and glutathione was shown to decrease mitochondrial transfer between mouse BMSCs and human primary AML cells and increase mouse survival significantly (31).

## Concluding remarks

**Figure 2:**
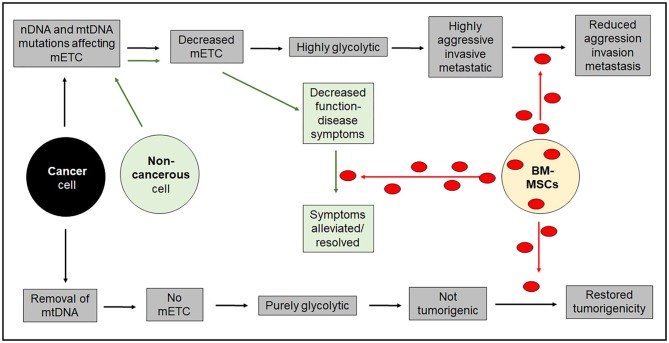
Diagrammatic representation of the long-term outcomes of intercellular mitochondrial transfer. Acquisition of mitochondria in non-cancerous cells that have defects in mitochondrial respiration are likely to benefit from acquisition of healthy mitochondria. The situation for cancer cells is more complicated and will depend on their ability to synthesize DNA. Highly aggressive cancer cells with diminished respiratory capacity will not benefit from acquiring healthy mitochondria that will impose a less aggressive phenotype. On the other hand purely glycolytic cancer cells that cannot synthesize DNA because of a block in pyrimidine biosynthesis in the complete absence of mitochondrial respiration will not be able to form tumors unless they acquire healthy mitochondria from the host. mETC = mitochondrial electron transport chain.

Intercellular mitochondrial transfer is a relatively new concept in tumor biology allowing replacement of mutated or treatment-damaged mitochondria in cancerous and non-cancerous cells. Harnessing the mitochondrial donating properties of cells in the body or following transplantation has the potential to be a game-changer in many diseases involving compromised mitochondrial function, including neurological and neuromuscular disorders and in aging, potentially leading to a lessening or even a reversal of disease symptoms. However, whether or not mitochondrial transfer could be an anti-cancer treatment remains to be seen. Highly aggressive cancer cells with mtDNA mutations that have acquired a highly aggressive invasive and metastatic phenotype would be ill-served by acquiring undamaged mitochondria from their environment. In those situations, mitochondrial transfer would be expected to decrease invasiveness and metastatic potential, and could be seen as a possible anti-cancer strategy. However, tumor cells without functional mtDNA due to severe deletions, that are unable to provide a source of oxidized ubiquinone for DHODH activity and are thus unable to synthesize pyrimidines to make DNA, need to acquire mitochondria from elsewhere to become established perhaps as secondary metastatic tumors. Figure 2 depicts the differential effects of intercellular mitochondrial transfer in different scenarios.

Although we can speculate about the benefits of mitochondrial transfer as a treatment option for cancer, we do not know the extent to which this is a physiological occurrence *in vivo*, nor do we know much about cells that have the potential to donate mitochondria in the body. Although BM-MSCs are the most widely used cell type to be used in mitochondrial transfer studies *in vitro*, skin fibroblasts and endothelial cell are also able to donate mitochondria under certain conditions. While directly correcting detrimental mitochondrial mutations may be an insurmountable treatment barrier with current technologies, exploiting mitochondrial movement between cells for health gain is a more tangible goal that could see translation into the clinic in the forseeable future. Although not all intercellular mitochondrial transfer will be compatible with nuclear genetics, even within a species, these challenges are potentially surmountable and could lead to a new wave of regenerative applications in the health and medical sciences.

## Author contributions

This invited review was conceived by MB and PH contributing equally to the writing. RD provided text in her areas of expertise. RD generated Figure 1 and PH generated Figure 2.

### Conflict of interest statement

The authors declare that the research was conducted in the absence of any commercial or financial relationships that could be construed as a potential conflict of interest.
